# Reliability of medical records in diagnosing inflammatory breast cancer in Egypt

**DOI:** 10.1186/s13104-017-2433-z

**Published:** 2017-03-16

**Authors:** Lynne Le, Catherine Schairer, Ahmed Hablas, Jane Meza, Shinobu Watanabe-Galloway, Mohamed Ramadan, Sofia D. Merajver, Ibrahim A. Seifeldin, Amr S. Soliman

**Affiliations:** 1 University of Nebraska Medical Center College of Public Health, 984395 Nebraska Medical Center, Omaha, NE 68198 USA; 2 National Cancer Institute, 9609 Medical Center Drive, Bethesda, MD 20892 USA; 3 Gharbiah Cancer Society, Tanta, Egypt; 4 University of Michigan Medical School, 1500 E Medical Center Dr, Ann Arbor, MI 48109 USA; 5 Department of Epidemiology, College of Public Health, University of Nebraska Medical Center, 984395 Nebraska Medical Center, Omaha, NE 68198-4395 USA

**Keywords:** Medical record, Egypt, North Africa, Inflammatory breast cancer

## Abstract

**Background:**

Inflammatory breast cancer (IBC) is a rare, aggressive breast cancer diagnosed clinically by the presence of diffuse erythema, peau d’orange, and edema that arise quickly in the affected breast. This study evaluated the validity of medical records in Gharbiah, Egypt in identifying clinical signs/symptoms of IBC. For 34 IBC cases enrolled in a case–control study at the Gharbiah Cancer Society and Tanta Cancer Center, Egypt (2009–2010), we compared signs/symptoms of IBC noted in medical records to those recorded on a standardized form at the time of IBC diagnosis by clinicians participating in the case–control study. We calculated the sensitivity and specificity of medical records as compared to the case–control study for recording these signs/symptoms. We also performed McNemar’s tests.

**Results:**

In the case–control study, 32 (94.1%) IBC cases presented with peau d’orange, 30 (88.2%) with erythema, and 31 (91.2%) with edema. The sensitivities of the medical records as compared to the case–control study were 0.8, 0.5, and 0.2 for peau d’orange, erythema, and edema, respectively. Corresponding specificities were 1.0, 0.5, and 1.0. p values for McNemar’s test were <0.05 for all signs. Medical records had data on the extent and duration of signs for at most 27% of cases for which this information was recorded in the case–control study. Twenty-three of the 34 cases (67.6%) had confirmed diagnosis of IBC in their medical records.

**Conclusion:**

Medical records lacked information on signs/symptoms of IBC, especially erythema and edema, when compared to the case–control study. Deficient medical records could have implications for diagnosis and treatment of IBC and proper documentation of cases in cancer registries.

## Background

Inflammatory breast cancer (IBC) is a rare and aggressive type of locally advanced breast cancer (LABC) characterized by diffuse erythema and edema (or peau d’orange) of the breast [1, 2]. The IBC diagnosis is primarily clinical but also requires pathological confirmation of cancer. It can be confused with other types of neglected LABC but is differentiated by the rapid onset of symptoms.

The definition of IBC according to the seventh edition of the American Joint Committee on Cancer (AJCC) Cancer Staging Manual has three requirements: (A) the presence of diffuse erythema, edema, and peau d’orange over at least one-third of the breast, and (B) rapid onset of symptoms, and; (C) a pathologic diagnosis of breast cancer. There is frequently no underlying tumor mass. Previous AJCC definitions of IBC have included variable proportions of breast involvement—from no specified proportion requirement (AJCC 5th edition) [3] to the “majority” of the breast (AJCC 6th edition) [4] to one-third of the breast (AJCC 7th edition) [5]. Some countries have used different definitions of IBC, such as “pousee evolutive,” which is used in Tunisia [6].

There is an urgent need to better identify this condition in population-based cancer registries around the world. In the surveillance, epidemiology and end results (SEER) database in the United States, IBC is defined by a combination of histology, stage, and Extent of Disease (EOD) codes, which accommodate changes in the AJCC definition over time. These codes are not routinely used in other population-based cancer registries around the world, including those in North Africa. Therefore, IBC is not routinely identified in most population-based cancer registries. Accurate registration of IBC in population-based registries requires both a set of codes to capture the clinical characteristics as well as complete and accurate recording of clinical signs and symptoms of IBC in routine medical records (MRs) and accommodating for different definitions to facilitate comparisons from different time periods and locations.

To our knowledge, there have been no previous studies in which the signs of IBC noted in medical records have been compared to signs/symptoms systematically recorded at the time of diagnosis by clinicians who have been specifically trained in the diagnosis of IBC (i.e., a gold standard). Therefore, this study aimed to evaluate the validity of medical records from the Nile Delta region of Egypt in the identification of IBC in the context of an ongoing case–control study.

## Methods

### Context: case–control study

Over the past 5 years, an epidemiologic case–control study was developed and conducted by several of the co-authors in Egypt, Tunisia, and Morocco to enroll IBC patients based on clinical evidence and investigate the epidemiology of the disease. The Tanta Cancer Center (TCC) and the Gharbiah Cancer Society (GCS) were the sources of patients for the Gharbiah study site in Egypt. Patients were recruited and enrolled if (A) they were female, Egyptian, and at least 18 years old; (B) had erythema and/or edema/peau d’orange in at least one-third of the breast; (C) did not have extensive ulceration; (D) had pathologic confirmation of cancer; (E) had no previous treatment for the present breast cancer, and; (F) had no history of breast cancer. The patients enrolled in this study were confirmed as IBC using the signs/symptoms present during clinical examinations in addition to pathological confirmation of cancer. During the period of 2009–2010, 40 patients were recruited from TCC and GCS and each case was given a unique study ID number.

### Data collection

As part of the case–control study, information on signs and symptoms associated with diagnosis of IBC was collected at the time of diagnosis on standardized forms based on the clinical examination by physicians participating in the case–control study. Information collected included the presence, extent, and duration of erythema, peau d’orange, and increase in breast size. In addition, information was collected on the following signs/symptoms of breast cancer that might be helpful in distinguishing IBC from other types of locally advanced breast cancer that are characterized by neglect and delayed diagnosis: the presence, size, and duration of a palpable mass, ulceration, and palpable axillary lymph nodes.

Abstraction forms were developed and used to identify possible IBC cases from medical records of the 2 study hospitals (TCC and GCS) during the same time period as the case–control study (2009–2010). The medical records collected included all patients who received neoadjuvant chemotherapy for breast cancer treatment at the same institutions. The same variables collected in the case–control study were included in the medical record abstraction.

### Data analysis

Data analysis focused on: (A) identifying the IBC cases included in the case–control study who had medical records available; (B) identifying the concordance between the signs/symptoms collected for the case–control study and the signs/symptoms recorded on the routine medical records; (C) calculating sensitivity and specificity for signs recorded in the medical records compared to those recorded for the case–control study, and; (D) performing McNemar’s tests for each symptom to determine if there was a significant difference between the two data sources, taking into account the paired nature of the data. A *p* value less than 0.05 suggested significant discordance. In this study, peau d’orange and edema were combined and analyzed together as peau d’orange. Similarly, erythema and inflammation were combined and analyzed as erythema. All statistical analysis was performed using SAS 9.2 (Cary, NC).

Medical records were available for 34 (85%) of the 40 cases that were recruited in the case–control study. The 6 remaining cases did not have medical records from GCS or TCC due to receiving treatment elsewhere. There were a number of additional variables abstracted from the medical records that were excluded from analysis in the present study, including the name of the examining physician for each patient, which is referenced in the discussion. The study was approved by the University of Nebraska Medical Center IRB Committee (Protocol 060-13-EX) and the Gharbiah Cancer Society IRB Committee in Egypt. No patients were approached for this study and the secondary analysis of data of the medical records did not include any data of children. The consent was waived by the IRB Committees listed above in Egypt and Nebraska because of the secondary analysis of the aggregate data of the study.

## Results

Twenty-three of the 34 cases (67.6%) had confirmed IBC diagnosis in their medical records. According to case–control study, 30 (88.2%) of the 34 patients recruited presented with peau d’orange, 30 (88.2%) presented with erythema, and 31 (91.2%) with an increase in breast size. In the medical records, 24 (70.6%) were recorded as having peau d’orange, 18 (52.9%) as having erythema, and 7 (20.6%) as having an increase in breast size (Table 1). Further, case–control data on the size/extent and duration of peau d’orange was available for 29 (85.3%) and 24 (70.6%) of the 34 cases, respectively, whereas the same information was available for only 8 (23.5%) and 9 (26.5%) of the 34 cases, respectively, in the medical records. For erythema, information on extent and duration was available for 30 (88.2%) and 23 (67.6%) cases, respectively. In the medical records, the same information was recorded for 7 (20.6%) and 16 (47.1%) cases. Data regarding the extent of increase in breast size were available for 31 (91.2%) cases; 24 (70.6%) cases had information on duration of increase in breast size. Only 1 case from the medical records had information on the extent of increase in breast size, and 7 (20.6%) cases had information on duration of the symptom.

**Table 1:** Comparison of information available on the presence, extent, and duration of peau d’orange and erythema in the case–control study and in medical records (N = 34)

	Case–control study	MR
Sign		
Peau d’orange^a,b^		
Presence	30 (88.2%)	24 (70.6%)
Extent	29 (85.3%)	8 (23.5%)
Duration	24 (70.6%)	9 (26.5%)
Erythema^a,c^		
Presence	30 (88.2%)	18 (52.9%)
Extent	30 (88.2%)	7 (20.6%)
Duration	23 (67.6%)	16 (47.1%)
Increase in breast size^a^		
Presence	31 (91.2%)	7 (20.6%)
Extent	31 (91.2%)	1 (2.9%)
Duration	24 (70.6%)	7 (20.6%)

This table excludes the 6 cases that lacked medical record data

^a^Symptoms are not mutually exclusive

^b^Peau d’orange and/or Edema

^c^Erythema and/or inflammation

As shown in Table 2, peau d’orange had the highest sensitivity (80%) of the 3 signs and edema the lowest (0.22). Peau d’orange and edema had specificities of 100%, but results were based on very small sample sizes. Peau d’orange, erythema, and increase in breast size all had significant p-values (0.014, 0.0027, p < 0.0001, respectively) for the McNemar’s test. Only 5 cases had complete information in the medical records on presence, extent, and duration of erythema, peau d’orange, and edema/increase in breast size.

**Table 2:** Comparison of symptoms required for IBC, as recorded in the case–control study (N = 34) versus in medical records for patients recruited in the NAS

	Peau d’orange^a^		Erythema^b^		Increase in breast size	
	Case–control study		Case–control study		Case–control study	
	Yes	No	Yes	No	Yes	No
Medical records						
Yes	24	0	16	2	7	0
No	6	4	14	2	24	3
	Sensitivity = 0.8		Sensitivity = 0.53		Sensitivity = 0.22	
	Specificity = 1.0		Specificity = 0.5		Specificity = 1.0	
	p = 0.014		p = 0.0027		p < 0.0001	

This table excludes the 6 cases from the case–control study that had no medical records

p-values are derived from McNemar’s tests

^a^ Edema and/or Peau d’Orange

^b^ Erythema and/or inflammation

Table 3 lists other signs that were also recorded in the case–control study. Presence of a palpable mass was recorded for 27 cases (79.4%) by clinicians participating in the case–control study, slight ulcerations for 5 cases (14.7%), and palpable axillary lymph nodes for 28 (82.4%) cases. Sensitivities for all three signs were around 0.60 and specificities especially high for ulceration (0.97) and palpable lymph nodes (0.83). McNemar’s test for presence of a palpable mass and ulceration were not statistically significant (0.07 and 0.56, respectively).

**Table 3:** Comparison of additional symptoms as recorded in the case–control study (N = 34) versus in medical records for the same patients

	Presence of palpable mass		Ulceration		Palpable axillary lymph nodes	
	Case–control study		Case–control study		Case–control study	
	Yes	No	Yes	No	Yes	No
Medical records						
Yes	16	4	3	1	16	1
No	11	3	2	28	12	5
	Sensitivity = 0.59		Sensitivity = 0.6		Sensitivity = 0.57	
	Specificity = 0.43		Specificity = 0.97		Specificity = 0.83	
	p = 0.07		p = 0.56		p = 0.0023	

This table excludes the 6 cases from the case–control study that had no medical records

p-values are derived from McNemar’s tests

## Discussion

Our results suggest that there are serious limitations in the recording of signs and symptoms needed for the diagnosis of inflammatory breast cancer according to AJCC [5, 7] criteria in the medical records of the Tanta Cancer Institute and the Gharbiah Cancer Society in Tanta, Egypt. This was particularly true for erythema and for the extent and duration of all three signs (erythema, peau d’orange, and increase in breast size).

There may be a variety of reasons why examining physicians would fail to report all IBC signs and symptoms in patient medical records. IBC is a rare type of breast cancer and is not well understood by physicians in many places. For example, some physicians lack education regarding IBC diagnoses, as is the case among primary care physicians in Egypt and Tunisia [8]. This could be due to a paucity of emphasis on oncology during medical school, the fragmentation of cancer-focused education at both the undergraduate and graduate level, and the absence of continuing medical education post-graduation [9, 10].

Previous studies have found that physicians often fail to obtain or access continuing medical education (CME) modules, especially in developing countries, which thereby affects their knowledge regarding new cancer epidemiology and accurate cancer diagnosis and management [8, 11–13]. CME is necessary to keep health professionals up to date as the IBC definition evolves. There also needs to be increased emphasis on the standardization of these cancer-focused education programs across institutions.

Strengths of this study included the use of a separate, ongoing case–control study as a gold standard. The case–control study was conducted by IBC-trained physicians who recruited patients based on a clinical exam and recorded thorough information on the signs and symptoms present using standardized forms, allowing for comparison with medical record data. Limitations of this study include the relatively small number of IBC cases and variability in diagnoses due to the number of physicians examining patients (N = 19, data not shown).

## Conclusions

This study revealed deficits in the information recorded on medical records needed to identify IBC cases. Deficient medical record information could negatively impact the diagnosis and treatment for IBC patients. Furthermore, deficient medical record information could affect the perceived prevalence of IBC and limit the ability to record IBC cases in cancer registries, which would thereby hinder the study of its management and prevention epidemiology. Our findings support the need for a universal definition of IBC to help improve the reliability of medical records for registry-based research or ways to accommodate for multiple definitions to facilitate IBC research in developing countries. Future studies should compare other Egyptian institutions as well as neighboring developing countries to determine the most appropriate method of maintaining medical records for reliably identifying IBC cases. Additionally, improvements in professional education regarding the symptoms and clinical characteristics of IBC would allow future professionals to correctly diagnose true IBC cases as such and stay up to date upon diagnostic criteria and state of the art research surrounding IBC. International registries should also explore a standardized procedure for identifying and registering IBC cases.

## Abbreviations

IBC: inflammatory breast cancer
LABC: locally advanced breast cancer
AJCC: The American Joint Committee on Cancer
SEER: surveillance, epidemiology and end results
EOD: extent of disease codes
TCC: Tanta Cancer Center
GCS: Gharbiah Cancer Society
CME: continuous medical education
MRs: medical records
SAS: statistical analysis software
